# A Multimodality Treatment Approach for the Management of Recurrent Cervical Cancer in an Elderly Female Patient: A Case Report

**DOI:** 10.7759/cureus.65424

**Published:** 2024-07-26

**Authors:** Manishimwe Jules, Neha Rahul, Induni N Weerarathna, Anurag Luharia

**Affiliations:** 1 Radiation Therapy, Datta Meghe Institute of Higher Education and Research, Wardha, IND; 2 Radiation Oncology, Datta Meghe Institute of Higher Education and Research, Wardha, IND; 3 Biomedical Sciences, Datta Meghe Institute of Higher Education and Research, Wardha, IND; 4 Radiotherapy, Datta Meghe Institute of Higher Education and Research, Wardha, IND

**Keywords:** brachytherapy, multimodal treatment, recurrence, squamous cell carcinoma, cervical cancer

## Abstract

Squamous cells in the cervix can develop into a type of cervical cancer. Cervical squamous cells are the cells that line the outside of the cervix. These thin, flat cells have a striking resemblance to fish scales under a microscope. Squamous cell carcinomas (SCCs) are the most common type of cervical cancer. We report the case of a 60-year-old woman with SCC devoid of a family history of cancer or related diseases. Following a biopsy confirming SCC, the patient's contrast-enhanced computed tomography scan revealed a somewhat enlarged cervix along with a white discharge per vagina. The patient underwent a Wertheim hysterectomy and was diagnosed with microinvasive SCC, adenomyosis, and negative lymph nodes. Two years after being free from disease, the issue reappeared even with routine follow-ups. The patient underwent six rounds of chemotherapy, followed by chemoradiation and interstitial brachytherapy. The multimodality therapy method applied to an aged female patient experiencing recurrent SCC of the cervix is demonstrated in this case study. It underlines how crucial regular follow-ups and multimodal therapy are to control recurrent cervical cancer.

## Introduction

Currently, cervical cancer is a significant health issue, especially in underdeveloped areas where access to consistent screening and early prevention may be limited [1,2]. Diagnostic tests assist in identifying bleeding, pelvic discomfort, and abnormal vaginal discharge [3]. This case study mostly follows a 60-year-old woman without any apparent cancer family history or comorbidities. Initially, she exhibited white per vaginal discharge in February 2021, and after investigation, she was diagnosed with squamous cell carcinoma (SCC) of the cervix. The patient underwent a Wertheim hysterectomy, but in August 2023, pelvic discomfort with white discharge per vaginal resurfaced.

Brachytherapy, radiation, and chemotherapy helped to inhibit the recurrence, therefore highlighting the need for a multimodal approach to recurrent cervical cancer [4,5]. From first diagnosis to surgical intervention, recurrence, and additional treatment, the patient's path displays the challenges in cervical cancer control. This scenario underlines the need for follow-up, the essential element of a good and correct diagnosis, and the effectiveness of integrated therapeutic modalities in enhancing patient outcomes [6]. This case helps clarify the challenges and achievements in treating recurrent cervical cancer, improving knowledge of its therapy.

## Case presentation

A 60-year-old female patient free of significant comorbidities or family history of cancer presented in February 2021 with a history of white discharge per vagina (PV). She had a biopsy on February 15, 2021, and found features connected to SCC. Her clinical per vaginal examination showed bulky cervical lips with smooth vaginal, rectal mucosa, and normal bilateral parametrium. Eight days later, the hospital conducted a contrast-enhanced CT (CECT) scan of the abdomen and discovered a somewhat big cervix with minimal homogenous enhancement. The fat planes connecting the cervix, urinary bladder (UB), and rectum were preserved. A minimum hypodensity in the endometrial canal indicated a probable collection. She was staged as FIGO IB1 based on clinical and radiological evaluation.

On March 3, 2021, the patient underwent a Wertheim hysterectomy; the histopathological report confirmed SCC of the cervix. The dimensions of the uterus were 8 × 4 × 2.5 cm with a 2 cm cervix. One section showed focal areas of in situ SCC with microinvasion. The myometrium revealed adenomyosis, while the endometrium, cervix, both parametrium, right ovary, left ovary, right uterine artery, right external iliac nodes, and left external iliac nodes were negative for malignancy. The lymph nodes examined were 0/8.

**Figure 1:**
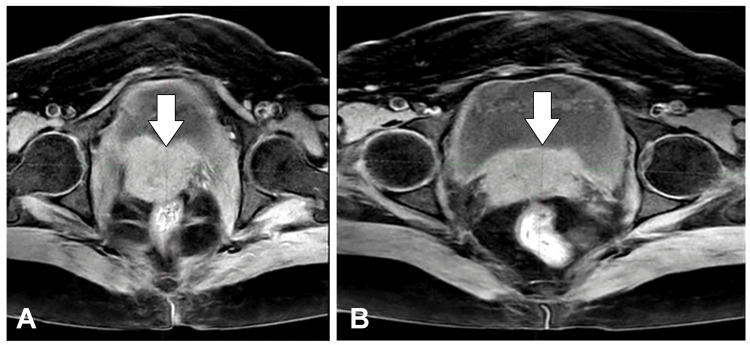
(A,B) Images of the T2-weighted MRI showing the recurrent disease in the vault of the patient (white arrows) MRI: magnetic resonance imaging

The patient remained on regular follow-up until August 2023, when she developed white discharge PV and pelvic pain. Investigations included a contrast-enhanced magnetic resonance imaging (CE MRI) of the pelvis, which revealed an enhancing lobulated irregular mass of approximately 6 × 6 × 4 cm in the cervix region, invading the posterior wall of the UB while maintaining fat planes with the rectum. A September 2, 2023, biopsy confirmed moderately differentiated SCC of the cervix. Figure 1 shows an image of the T2-weighted MRI showing the recurrent disease in the vault of the patient.

**Figure 2:**
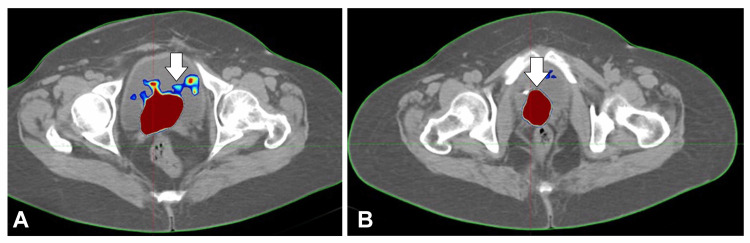
(A,B) Images of the fused PET scan with MRI showing the recurrent disease in the vault of the patient (white arrows) PET: positron emission tomography; MRI: magnetic resonance imaging

A positron emission tomography (PET) scan on September 5, 2023, showed posthysterectomy status with a fluorodeoxyglucose avid irregular mixed density soft-tissue mass involving the vaginal vault, measuring about 4.2 × 3.7 cm, with a SUVmax of 15. The mass invaded the posterior wall of the UB and encased the distal left ureter, resulting in moderate right hydroureteronephrosis. Serum creatinine on August 31, 2023, was 0.8 mg/dL. The images of the fused PET scan with MRI show the recurrent disease in the vault of the patient (Figure 2).

On examination at the Radiation Oncology OPD, the patient's general condition was good, with a Karnofsky performance status of 80. No significant clinical findings were noted. A pelvic examination revealed a vault mass at the 10 to 1 o'clock position, 4 cm from the vaginal introitus, with anterior vaginal wall involvement, while the rest of the vagina was free. Bilateral parametrium was involved medially, and the rectal mucosa was free.

**Figure 3:**
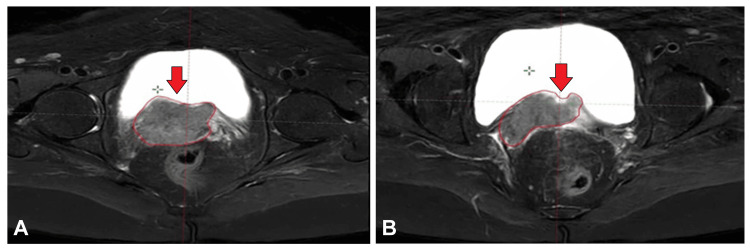
(A,B) Images of the T2-weighted MRI showing the residual disease in the vault of the patient after completing six cycles of systemic chemotherapy (red arrows) MRI: magnetic resonance imaging

The patient received six cycles of chemotherapy with paclitaxel and carboplatin at the hospital. There was mild regression in the lesion size with similar parametrial disease. A follow-up CECT of the abdomen on December 7, 2023, showed a tiny hepatic cyst in segment VII, mild dilatation of the right renal pelvis with a double-J stent, and subcentimetric mesenteric lymphadenopathy. Figure 3 displays the images of the T2-weighted MRI, showing the residual disease in the vault of the patient after completing six cycles of systemic chemotherapy.

CECT of the thorax on December 7, 2023, revealed dependent ground-glass opacities in both lungs' posterior and posterior-basal segments, along with mild emphysematous changes in the right lower lobe, which were suggestive of infective changes. The patient's general condition remained good on further examination, with a KPS of 90. No significant findings were noted in the supraclavicular fossa or abdomen. Pelvic examination after external beam radiotherapy (EBRT) indicated residual disease at the vault apex, predominantly on the right side, measuring 2 × 1 cm. The rest of the vaginal walls were smooth, and the vaginal length was 6 cm with a diameter of 2 cm. The right parametrium was involved in the medial two-thirds, while the left parametrium and rectal mucosa were free.

**Figure 4:**
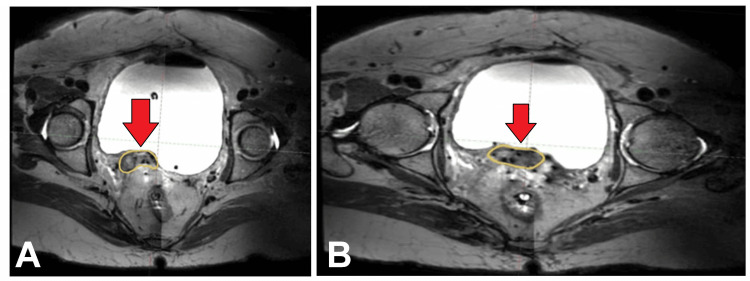
(A,B) Images of the T2-weighted MRI showing the residual disease in the vault of the patient after completing external beam radiotherapy (red arrows) MRI: magnetic resonance imaging

**Figure 5:**
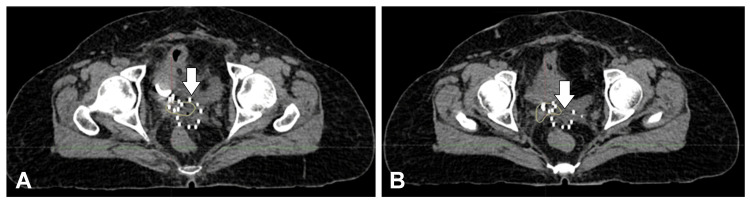
(A) An image of the interstitial brachytherapy application on the patient using Kelowna applicator and interstitial needles (white arrow). (B) An image of the planning CT scan for interstitial brachytherapy showing the interstitial needles (white arrow) CT: computed tomography

**Figure 6:**
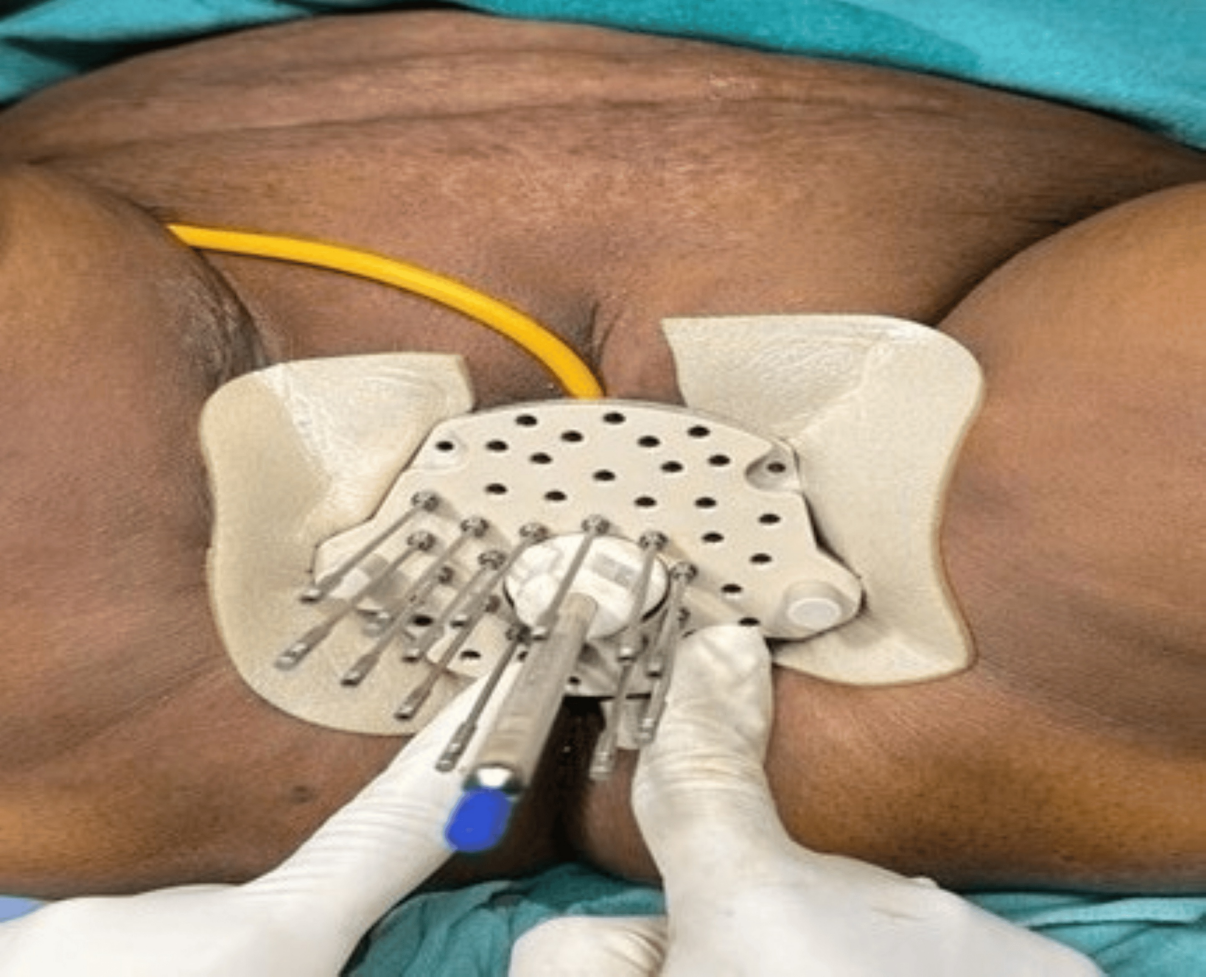
An image of the interstitial brachytherapy application on the patient using Kelowna applicator and interstitial needles

The patient completed definitive chemoradiotherapy to the whole pelvis to a dose of 50 Gy in 25 fractions using 3D conformal radiotherapy for one month and four cycles of concurrent chemotherapy. She tolerated the treatment well. Post-EBRT evaluation using an MRI pelvis dated February 26, 2023, revealed residual disease, and the patient was planned for interstitial brachytherapy to a dose of 5 Gy in four fractions using a Kelowna applicator with interstitial needles. Figure 4 shows an image of the T2-weighted MRI showing the residual disease in the vault of the patient after completing EBRT. Figure 5 shows the images of the planned CT scan for interstitial brachytherapy showing the interstitial needles. Figure 6 shows an image of the interstitial brachytherapy application on the patient using the Kelowna applicator and interstitial needles.

## Discussion

Cervical cancer is a leading cause of cancer-related deaths in women globally. Cervical cancer screening is crucial for women to detect preinvasive illness before it progresses to the invasive stage. Risk factors are assessed, and screening methods are chosen based on age, screening history, and available resources [7]. Recurrent cervical cancer management depends on previous treatment approaches, location, and the extent of recurrence and should be addressed by a multidisciplinary team [8].

The offered situation shows the challenges and nuances of managing recurrent cervical cancer. Early surgery and continuous follow-up still revealed the aggressive character of SCC and the need for constant monitoring as the patient had a recurrence [9]. Recurrence was treated with radiation used in a multimodal method, interstitial brachytherapy, and chemotherapy. With this all-encompassing treatment approach, tumor management should be maximized while the patient's quality of life is preserved [10]. Modern imaging techniques define the degree of the disease and directly inform therapy options, including CECT, PET scans, and CE MRI. The choice to proceed with interstitial brachytherapy emphasizes the great need to precisely treat the residual tumor using high-dose radiation to target malignant cells and protect healthy tissues [11,12].

This example emphasizes the need to use a multimodal approach to treat recurrent cervical cancer since many cases show significant anatomical and therapeutic challenges. Radiation, chemotherapy, surgery, and better imaging integrated into a multimodal strategy define the treatment of complex diseases and improve patient outcomes.

## Conclusions

This case report shows the entire therapeutic course used for managing recurrent cervical cancer involving surgery, chemotherapy, radiation therapy and interstitial brachytherapy. It emphasizes good imaging, frequent follow-up, and quick responsiveness. The therapy approach chosen dramatically improves patient outcomes and disease control. This situation also emphasizes the vital requirement of ongoing research and inventiveness in the treatment of recurrent cervical cancer to raise survival rates and quality of life for affected persons.
